# Facial discoid dermatosis: A cosmetically disfiguring and challenging condition to treat

**DOI:** 10.1002/ski2.56

**Published:** 2021-06-24

**Authors:** S. Rahmatulla, K. Batta, F. Tatnall, D. Sandhu, V. Brown

**Affiliations:** ^1^ Department of Dermatology West Hertfordshire Hospitals NHS Trust Watford Hertfordshire UK; ^2^ School of Life and Medical Sciences University of Hertfordshire Hatfield Hertfordshire UK

## Abstract

Facial discoid dermatosis (FDD) is a recently described condition comprising discrete facial papulo‐squamous lesions. We report three cases that clinically and histologically resemble FDD and demonstrate its resistance to treatment. Awareness of this new clinical entity will allow early diagnosis and the ability to make patients aware that there is unlikely to be a successful treatment. However, our study suggests that although FDD can persist for many years, it appears to remain stable.

## Graphical abstract

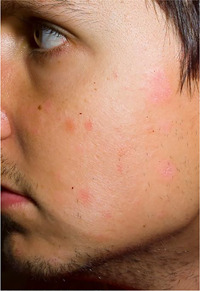

1

**What is already known about this topic?**

- Facial Discoid Dermatoses presents as papulosquamous lesions that localise to the face; it is resistant to all therapies.

**What does this study add?**

- Increases the awareness of this condition so that earlier diagnosis can be made.

## 2 INTRODUCTION

We have seen three cases with multiple persistent discoid well‐defined psoriasiform lesions on the face that do not fit a well‐known clinical or histological diagnosis and do not respond to a myriad of therapies. The entity facial discoid dermatosis (FDD) has been suggested for this group of disorders.^1^ It presents as discrete thin papulo‐squamous pink‐orange lesions that localise to the face, sometimes accompanied with scale, that are highly resistant to treatment, though remain stable for many years. Awareness of this new clinical entity will allow early diagnosis of patients who have similar persistent facial lesions. With increased reporting of this rare entity, there will be progress in further defining its characteristics and management strategies.

## 3 REPORT

**FIGURE 1:**
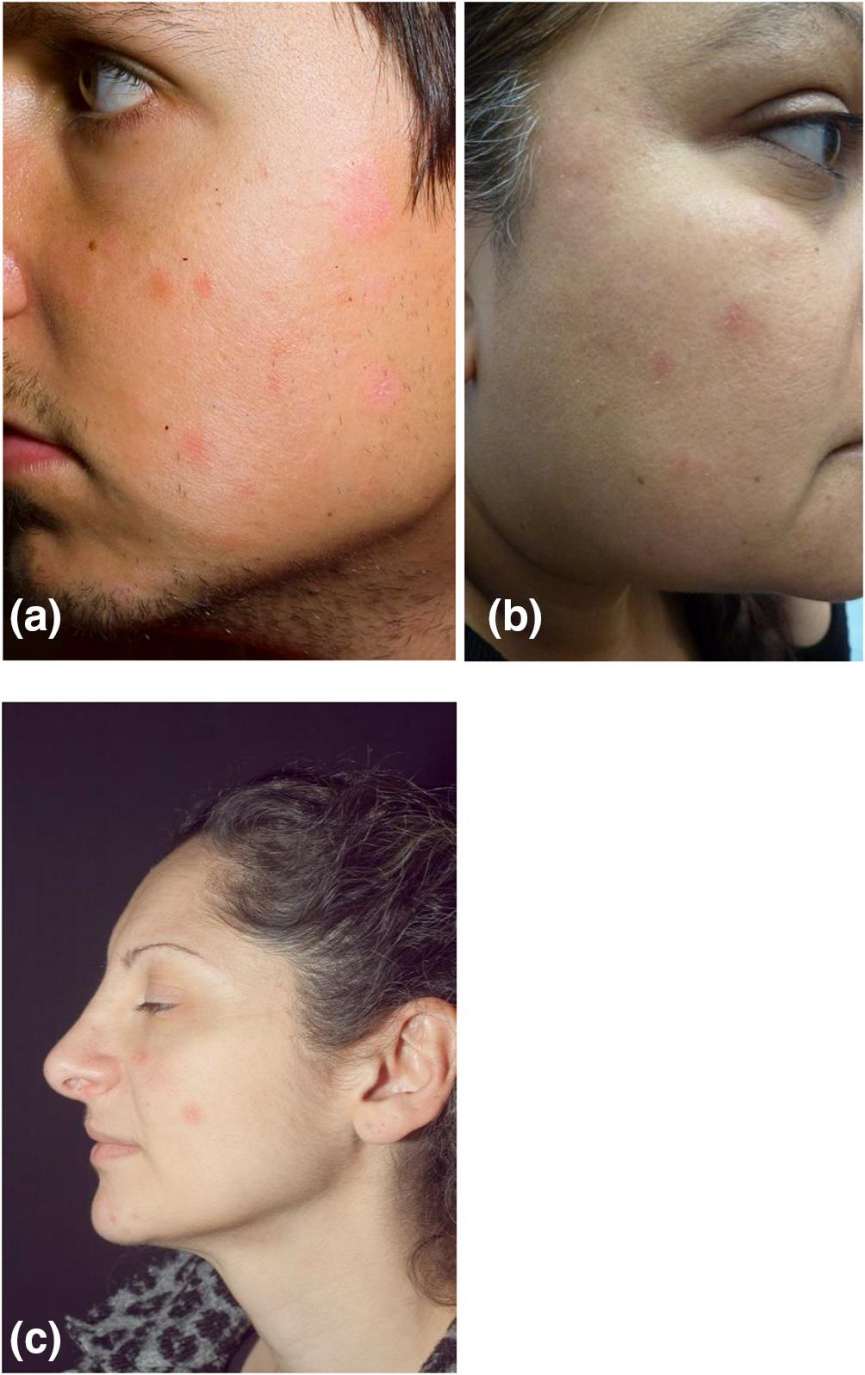
(a) Case 1 (b) Case 2 (c) Case 3. All three cases showed well‐defined facial erythematous plaques

**FIGURE 2:**
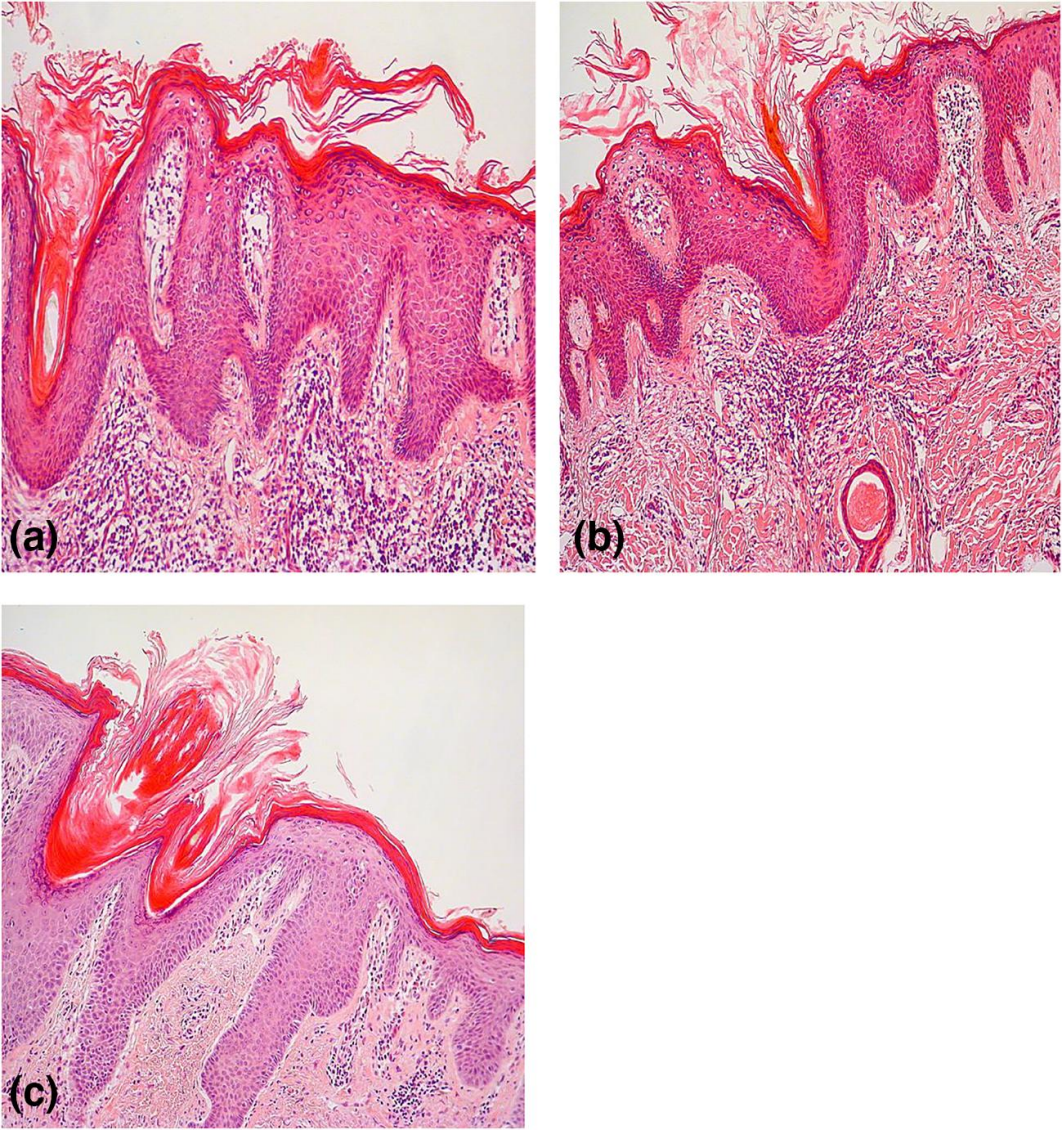
Haematoxylin and eosin staining (a) Case 1, hyperkeratosis, parakeratosis, acanthosis, follicular plugging and a lymphocytic infiltration in the superficial dermis, (b) Case 2, hyperkeratosis, acanthosis and a dermal lymphocytic infiltrate (c) Case 3, hyperkeratosis, focal parakeratosis, acanthosis, follicular plugging and a dermal lymphocytic infiltrate

Case 1 A 19‐year‐old male with Fitzpatrick Skin Type 2 presented with an 18‐month history of an asymptomatic facial rash. Antecedent treatments included coal tar, hydrocortisone and allantoin cream and Clotrimazole 1% cream. On examination he had discrete orange‐pink slightly scaly discoid plaques on the cheeks and forehead (Figure 1a). He was clinically diagnosed with sebopsoriasis however failed to respond to clobetasone 17‐butyrate 0.05% w/w, oxytetracycline 3.0% w/w as calcium oxytetracycline and nystatin 100 000 units per gram cream, clobetasone butyrate 0.05% ointment, mometasone furoate 0.1% ointment, clobetasol propionate 0.05% ointment, ketoconazole 2% shampoo, tacrolimus 0.1% ointment, pimecrolimus 1% cream and tacalcitol ointment. Systemic itraconazole 200 mg once a day for 4 weeks and oxytetracycline 500 mg twice daily for 8 weeks was also trialled without response. A biopsy showed hyperkeratosis, parakeratosis, acanthosis, follicular plugging and a lymphocytic infiltration in the superficial dermis (Figure 2a). Staining for periodic acid‐Schiff (PAS) was negative. On the basis that the rash may represent psoriasis, the patient was treated with Narrowband UVB phototherapy (NBUVB) and Acitretin 25 mg once a day for 8 weeks but the facial plaques persisted. A trial of hydroxychloroquine 200 mg twice daily for 12 weeks for a presumed clinical diagnosis of discoid lupus erythematosus (DLE) gave no clinical improvement. This was followed by cryotherapy and five sessions of pulsed dye laser which was also ineffective. His lesions have persisted but have remained stable in number and extent without progression during a follow up period of 8 years.

Case 2 A 40‐year‐old female with Fitzpatrick Skin Type 4 presented with a 7‐year history of multiple asymptomatic facial erythematous plaques, which had been treated with miconazole nitrate with hydrocortisone cream with no benefit. On examination, she had multiple small discoid erythematous plaques on her face (Figure 1b). Differential diagnoses included DLE and psoriasis. Histological examination from one of the plaques showed hyperkeratosis, acanthosis and a dermal lymphocytic infiltrate (Figure 2b). Staining for PAS was negative. She was treated for a presumed clinical diagnosis of DLE, however, failed to respond to topical steroids (mometasone furoate 0.1% ointment and clobetasol propionate 0.05% ointment), tacrolimus 0.1% ointment and a 6‐month course of hydroxychloroquine 200 mg twice daily. A repeat biopsy showed some psoriasiform changes, and therefore, she received a course of NBUVB followed by calcipotriene ointment which was ineffective.

Case 3 A 44‐year‐old female with Fitzpatrick Skin Type 3 presented with asymptomatic facial plaques which had gradually increased in number and had been unresponsive to mildly potent topical corticosteroids. She had a history of depression treated with fluoxetine. Examination revealed 4 mildly scaly erythematous discoid plaques on the face (Figure 1c). Skin scraping was negative for mycology. Given the clinical suspicion of DLE, a skin biopsy was performed which showed hyperkeratosis, focal parakeratosis, acanthosis, follicular plugging and a dermal lymphocytic infiltrate (Figure 2c). Treatment for presumed DLE with mometasone furoate 0.1% ointment, clobetasol propionate 0.05% ointment and hydroxychloroquine 200 mg twice a day for 6 months was unsuccessful. She was diagnosed with FDD and, recognising the resistance of this condition to treatment, she was not offered any further therapeutic options.

## 4 DISCUSSION

FDD was first reported in 2010. It is an under recognised condition characterised by persistent orange‐pink annular plaques, with variable surface scale, that localise to the face and neck.^1^

Multiple therapies have been trialled without success in the reports published to date.^1^, ^2^, ^3^, ^4^ Topical treatments include potent and very potent topical corticosteroids, vitamin D analogues, betamethasone/calcipotriene ointment, antifungals, calcineurin inhibitors, tazarotene cream, tretinoin 0.05% cream and imiquimod cream. Oral treatments include doxycycline, itraconazole, acitretin, hydroxychloroquine, prednisolone and methotrexate. Other treatments include intralesional steroids, NBUVB and Pulsed dye laser.

Histologically, FDD demonstrates hyperkeratosis, parakeratosis, acanthosis and follicular plugging. A lymphocytic infiltrate in the superficial dermis is also commonly seen.^1^, ^2^, ^3^, ^4^ In the largest case series of eight patients, additional histological features of subcorneal acantholysis and checkerboard alternating orthokeratosis and parakeratosis were seen.^2^ The authors, therefore, postulate that FDD may be a new variant of pityriasis rubra pilaris. One of these cases went on to develop Type 2 pityriasis rubra pilaris.^2^ However, as in our case series, none of the other reported cases developed extra‐facial disease. In all reported cases to date, the condition has persisted without progression or resolution, suggesting that FDD is a novel clinical entity.

## CONFLICTS OF INTEREST

None to declare.

## Data Availability

The data that support the findings of this study are available from the corresponding author upon reasonable request.
